# Redox activity of thioredoxin *z* and fructokinase-like protein 1 is dispensable for autotrophic growth of *Arabidopsis thaliana*

**DOI:** 10.1093/jxb/eru122

**Published:** 2014-03-22

**Authors:** Matthias Wimmelbacher, Frederik Börnke

**Affiliations:** ^1^Friedrich-Alexander-Universität, Department of Biology, Division of Biochemistry, Staudtstr. 5, 91058 Erlangen, Germany; ^2^Leibniz-Institute of Vegetable and Ornamental Crops (IGZ), Theodor-Echtermeyer-Weg 1, 14979 Großbeeren, Germany; ^3^Institute of Biochemistry and Biology, University of Potsdam, 14476 Potsdam, Germany

**Keywords:** Chloroplast transcription, FLN1, plastid-encoded RNA polymerase, redox regulation, thioredoxins, TRX z.

## Abstract

Redox modulation of protein activity by thioredoxins (TRXs) plays a key role in cellular regulation. Thioredoxin *z* (TRX *z*) and its interaction partner fructokinase-like protein 1 (FLN1) represent subunits of the plastid-encoded RNA polymerase (PEP), suggesting a role of both proteins in redox regulation of chloroplast gene expression. Loss of TRX *z* or FLN1 expression generates a PEP-deficient phenotype and renders the plants incapable to grow autotrophically. This study shows that PEP function in *trx z* and *fln1* plants can be restored by complementation with redox-inactive TRX *z* C_106_S and FLN1 C_105/106_A protein variants, respectively. The complemented plants showed wild-type levels of chloroplast gene expression and were restored in photosynthetic capacity, indicating that redox regulation of PEP through TRX *z*/FLN1 *per se* is not essential for autotrophic growth. Promoter–reporter gene studies indicate that *TRX z* and *FLN1* are expressed during early phases of leaf development while expression ceases at maturation. Taken together, our data support a model in which TRX *z* and FLN1 are essential structural components of the PEP complex and their redox activity might only play a role in the fine tuning of PEP function.

## Introduction

Due to their sessile life style, plants have evolved a multitude of regulatory mechanisms that enable them to maintain their cellular homeostasis under constantly fluctuating environmental conditions. Redox reactions are central to photosynthetic energy conversion and critical for the redox state of a cell, which makes redox regulation a key element in adjusting plant metabolism and development to the prevailing environmental conditions (Foyer *et al.*, 2009). Redox modification of proteins is one route through which redox signals can be translated into cellular responses. Depending on their oxidation state, cysteine residues within proteins can form inter- or intramolecular disulphide bonds which can profoundly influence protein conformation and thus activity. In general, redox modifications of cysteine residues are controlled by small disulphide oxidoreductases named thioredoxins (TRXs) and glutaredoxins (Schürmann and Buchanan, 2008; Meyer *et al.*, 2009; König *et al.*, 2012).

Characteristic to TRXs is a redox-active disulphide bridge within a conserved amino acid sequence CXXC (where X indicates a variable residue), which is involved in thiol/disulphide exchanges with their target proteins. In most organisms, TRXs are encoded by a gene family that is particularly expanded in higher plants. The *Arabidopsis thaliana* genome codes for more than 20 typical TRX genes and about 30 TRX-like proteins (Meyer *et al.*, 2007, 2012). TRXs can be grouped according to their subcellular localization into cytosolic, plastidial, and mitochondrial isoforms. The complexity is particularly high in plastids, which contain 10 TRX isoforms. Based on their sequence similarity, these can be further subdivided into five groups consisting of four *m*-type, two *f*-type, two *y*-type, one *x*-type, and one *z*-type TRXs (Meyer *et al.*, 2005, 2012; Arsova *et al.*, 2010). This multiplicity of TRX isoforms in the same cellular compartment raised the question about their functional redundancy or possible specialization. While *f*- and *m*-type TRXs have mainly been implicated in the regulation of photosynthetic carbon metabolism (Buchanan and Balmer, 2005; Meyer *et al.*, 2005), TRXs *y* and *x* appear to be more specifically involved in the response to oxidative stress, in particular through the regeneration of antioxidant enzymes such as peroxiredoxins and methionine sulphoxide reductases (Collin *et al*., 2003, 2004).

TRX *z* has initially been identified as a component of plastid transcriptionally active chromosome (pTAC) from *Arabidopsis*, which represents a high-*M*_r_ DNA/RNA–protein complex containing approximately 40–60 proteins and that is capable of *in vitro* transcription (Pfalz *et al.*, 2006). Recently, several independent studies could demonstrate that TRX *z* constitutes a subunit of the plastid-encoded RNA polymerase (PEP) and thus likely functions in plastid transcription (Arsova *et al.*, 2010; Schröter *et al.*, 2010; Steiner *et al.*, 2011). Due to their endosymbiotic origin, plastids possess a reduced genome, the plastome, and a complete machinery to express the genetic information contained on it. Transcription of the plastome-encoded genes depends on two types of RNA polymerases: (1) a phage-type, single-subunit, nucleus-encoded plastid RNA polymerase (NEP); and (2) the prokaryotic-type, multisubunit PEP (Shiina *et al.*, 2005; Zubo *et al.*, 2011). PEP is composed of the plastome-encoded subunits α_2_, β, β′, and β′′ and, together with nuclear-encoded sigma factors, it mediates expression of genes mainly associated with the photosynthetic apparatus. In early stages of chloroplast development, PEP structure is most similar to that found in bacteria. However, in maturing chloroplasts, the core enzyme assembles with additional nuclear-encoded proteins into a multienzyme complex (Pfannschmidt *et al.*, 2000). These additional subunits likely expand PEP functionality and regulation in order to suit the specific functions of the photosynthetically active chloroplast (Link, 1996; Pfannschmidt and Liere, 2005). The PEP complex represents a major target for photosynthetic redox signals that allow the adjustment of plastid gene expression in response to unbalanced excitation of both photosystems (Baginsky *et al.*, 1999). The signalling cascades involved appear mainly to proceed via phosphorylation; however, recent evidence suggests interaction with a thiol-dependent signal (Steiner *et al.*, 2009). TRX *z* is a likely candidate for mediating thiol-modifications within the multisubunit PEP complex in chloroplasts. A yeast two-hybrid screen identified two fructokinase-like proteins (FLN1 and FLN2) as possible TRX *z* targets, and light-dependent reduction of FLN2 by TRX *z* could be demonstrated *in vivo* (Arsova *et al.*, 2010). In complementary studies, both FLN proteins were identified as intrinsic subunits of the PEP complex using proteomics (Schröter *et al.*, 2010; Steiner *et al.*, 2011). The *Arabidopsis* knockout mutant lines of TRX *z* and FLN1 exhibit a pale-yellow phenotype and can only grow heterotrophically on sucrose-supplemented medium (Arsova *et al.*, 2010; Schröter *et al.*, 2010; Steiner *et al.*, 2011). Gene expression analyses of the *trx z Arabidopsis* mutant revealed changes in plastid gene expression that are diagnostic for PEP deficiency, arguing for an essential role of TRX *z* in PEP function. In this respect, TRX *z* stands out from other TRXs that appear to have at least overlapping functions, as knockout of individual TRX genes results in only subtle phenotypic changes. In turn, a loss-of-function mutation of other nuclear-encoded PEP subunits frequently also results in a PEP-deficient phenotype associated with the inability to grow autotrophically and the characteristic changes in plastid gene expression (Pfalz and Pfannschmidt, 2013). The drastic phenotype of these mutants often led to the conclusion that each individual component has an essential regulatory role in PEP-mediated gene expression. However, an alternative explanation has recently been put forward emphasizing that a disturbance in the correct RNA polymerase complex formation during early plastid development is the primary defect causing the observed phenotype (Pfalz and Pfannschmidt, 2013). According to this model, the generation of the full PEP complement is an essential early step during chloroplast biogenesis and its disturbance cannot be reversed if a certain time point in the program has been passed. Thus, PEP-associated nuclear-encoded proteins would possess a structural role in the assembly of the PEP complex rather than an essential role in its regulation imparted by the particular biochemical activity of a given subunit.

The present study challenged this hypothesis by transforming redox-inactive variants of *TRX z* and FLN1 into respective *Arabidopsis* knockout mutant backgrounds, assuming that these proteins are devoid of a redox-regulatory function. The results indicate that, under standard growth conditions, these redox-inactive protein variants are fully sufficient to restore PEP function during early chloroplast development, supporting a model in which the structural integrity of the PEP complex constitutes a bottleneck for chloroplast maturation.

## Materials and methods

### Recombinant protein expression in *Escherichia coli* and TRX *z* activity assay

Expression and purification of recombinant TRX *z* and its variants was carried out as described (Arsova *et al.*, 2010). Thioredoxin activity was assessed using the insulin-reduction assay (Holmgren, 1979) with 5 μg purified His_6_-tagged recombinant protein lacking the predicted chloroplast-targeting peptide.

### Plant growth conditions

*A. thaliana* seeds were sown on Murashige and Skoog (MS) agar (Sigma-Aldrich) supplemented with 2% (w/v) sucrose and cultivated in tissue culture under a 16/8 light/dark regime (irradiance 150 μmol quanta m^–2^ s^–1^) with 50% relative humidity. *Arabidopsis* ecotype Columbia (Col) was used in all experiments. The single insert *fln1 Arabidopsis* T-DNA insertion mutant (GK_443A08) was obtained from the Nottingham *Arabidopsis* Stock Center (Nottingham University) and confirmed by PCR using the primers listed in Supplementary Table S1 (available at *JXB* online).

For cultivation on soil, wild-type *A. thaliana* plants and T-DNA insertion lines were stratified at 4 °C in the dark for 3 d. Subsequently, plants were grown under a 8/16 light/dark regime (22/20 °C) with 50% relative humidity and irradiance 100 μmol m^–2^ s^–1^.

### 35S:*FLN1* plasmid construction and plant transformation

The entire coding region of *FLN1* was amplified by PCR from *Arabidopsis* cDNA using the primers indicated in Supplementary Table S1, available at *JXB* online. The PCR fragment was inserted into the vector pENTR-D/TOPO (Life Technologies) and subsequently recombined into the binary vector pK7FWG2, which generates a C-terminal fusion with green-fluorescent protein (Karimi *et al.*, 2002). The resulting construct was designated 35S:*FLN1*–GFP and used to transform heterozygous *FLN1/fln1 Arabidopsis* plants by floral dip transformation (Clough and Bent, 1998).

### Site-directed mutagenesis

Site-directed mutagenesis of *TRX z* and *FLN1* constructs was performed using the Quick-Change site-directed mutagenesis kit (Agilent Technologies) employing the primers listed in Supplementary Table S1, available at *JXB* online. The pENTR–*TRX z* and pENTR-*FLN1* vectors were used as a template in the reaction and all base changes were verified by sequencing.

### Quantitative real-time PCR

For quantitative real-time reverse-transcription (qRT-PCR) analysis, total RNA was isolated from frozen plant material using the method described by Logemann *et al.* (1987). cDNA was synthesized and qRT-PCR was performed as previously described (Arsova *et al.*, 2010) on a Mx3000P Q-PCR system (Stratagene) in combination with the Brilliant II SYBR Green Q-PCR Master Mix Kit (Stratagene). Oligonucleotides used for determination of relative mRNA abundance of the different genes are summarized in Supplementary Table S1, available at *JXB* online.

### Measurement of photosynthesis parameters

Chlorophyll fluorescence imaging analysis was performed with a chlorophyll imaging system (MINI-Imaging-PAM Chlorophyll Fluorometer, Walz). The photosynthetic parameters were determined as described in Horst *et al.* (2008). Before each measurement, plants were dark adapted for 20min.

### Western blotting

Leaf material was homogenized in sodium dodecylsulphate polyacrylamide gel electrophoresis (SDS-PAGE) loading buffer (100mM Tris-HCl, pH 6.8; 9% β-mercaptoethanol, 40% glycerol, 0.0005% bromophenol blue, 4% SDS) and, after heating for 10min at 95 °C, subjected to gelectrophoresis. Separated proteins were transferred onto nitrocellulose membrane (Porablot, Machery and Nagel, Düren, Germany). Proteins were detected using an anti-GFP antibody (Roche) via chemiluminescence (GE Healthcare).

### Microscopy

Subcellular localization of the FLN1–GFP fusion protein in the p35S:*FLN1*–GFP/*fln1* plants was detected by confocal laser scanning microscopy (TCS SP5 II (AOBS), Leica Microsystems).

### Generation and analysis of GUS reporter lines

Promoter fragments comprising 1.5kb upstream of the start codon of the *TRX z* or *FLN1* coding region were amplified by PCR using genomic DNA from *Arabidopsis* Col-0 as a template and the primers listed in Supplementary Table S1, available at *JXB* online. The resulting fragments were cloned into the vector pENTR-D/TOPO (Life Technologies) and sequence verified. Subsequently, the fragments were fused with the GUS reporter gene in the vector pGWB3 using Gateway Cloning (Life Technologies).

*A. thaliana* Col-0 plants were transformed using standard procedures (Clough and Bent, 1998). After selection of transformed plants using BASTA treatment, the presence of the transgene was verified by PCR using *GUS*-specific primers. Expression of the reporter gene was monitored in T0, T1, T2, and/or T3 transgenic plants, using histochemical staining according to Jefferson *et al.* (1987).

## Results

### Mutation of either of the two cysteine residues within the catalytic site abolishes the biochemical activity of TRX *z*

Previously, Arsova *et al.* (2010) demonstrated that TRX *z* is a redox-active TRX. The protein contains the typical TRX active site C_106_GPC_109_ in which the two cysteine residues form the catalytically active disulphide bridge. Yeast two-hybrid analyses indicated that TRX *z* interacts with its FLN partner proteins in a TRX typical manner: the first cysteine in the catalytic site (position 106 according to the amino acid sequence of TRX *z*) forms a mixed disulphide bridge with the interaction partner, while the second sulphydryl residue rapidly reduces this disulphide bridge, releasing oxidized TRX *z* and reduced target protein. Therefore, a C_106_S mutation abolishes the interaction of TRX *z* with FLN1 and FLN2 while a C_109_S substitution has no effect on the interaction (Arsova *et al.*, 2010).

To test whether the loss of either one of the two cysteine residues affects the redox activity of the protein, this work generated TRX *z* variants by substituting either of the cysteines within the active sites by a serine residue. A mutated version of TRX *z* lacking the first cysteine in the redox active site is unable to form a mixed disulphide bridge with its substrate and therefore should also loose its redox activity. The protein variants were expressed in *E.coli* and recombinant His_6_-tagged TRX *z* (lacking the chloroplast transit peptide) was used to test the ability of disulphide-bridge reduction in an insulin-reduction assay (Holmgren, 1979). TRX reduces the intermolecular disulphide bonds between the insulin A and B chains and precipitation of the insoluble B chain can be measured photometrically by an increase in the absorbance at 650nm.

**Fig. 1.**
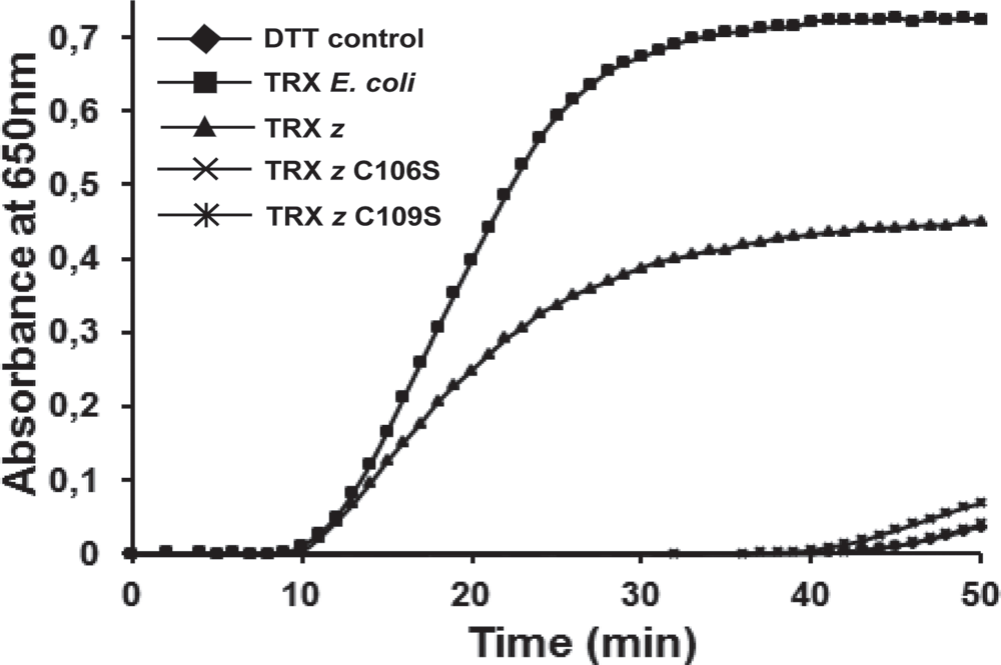
Measurement of disulphide reductase activity of recombinant TRX *z*. The insulin reduction assay was performed by using 5μM recombinant protein lacking the predicted chloroplast targeting peptide. *E. coli* TRX served as a positive control, while the nonenzymic insulin reduction by DTT served as a negative control.

As shown in Fig. 1, loss of either one of the two cysteines of the redox-active site leads to complete abolishment of insulin reduction activity of TRX *z* while the wild-type TRX *z* displayed disulphide reductase activity *in vitro*. This indicates that both cysteine residues of the redox-active site are essential for oxidoreductase activity of TRX *z*.

### A catalytically inactive TRX *z* variant is able to complement the *Arabidopsis trx z* knockout phenotype

**Fig. 2.**
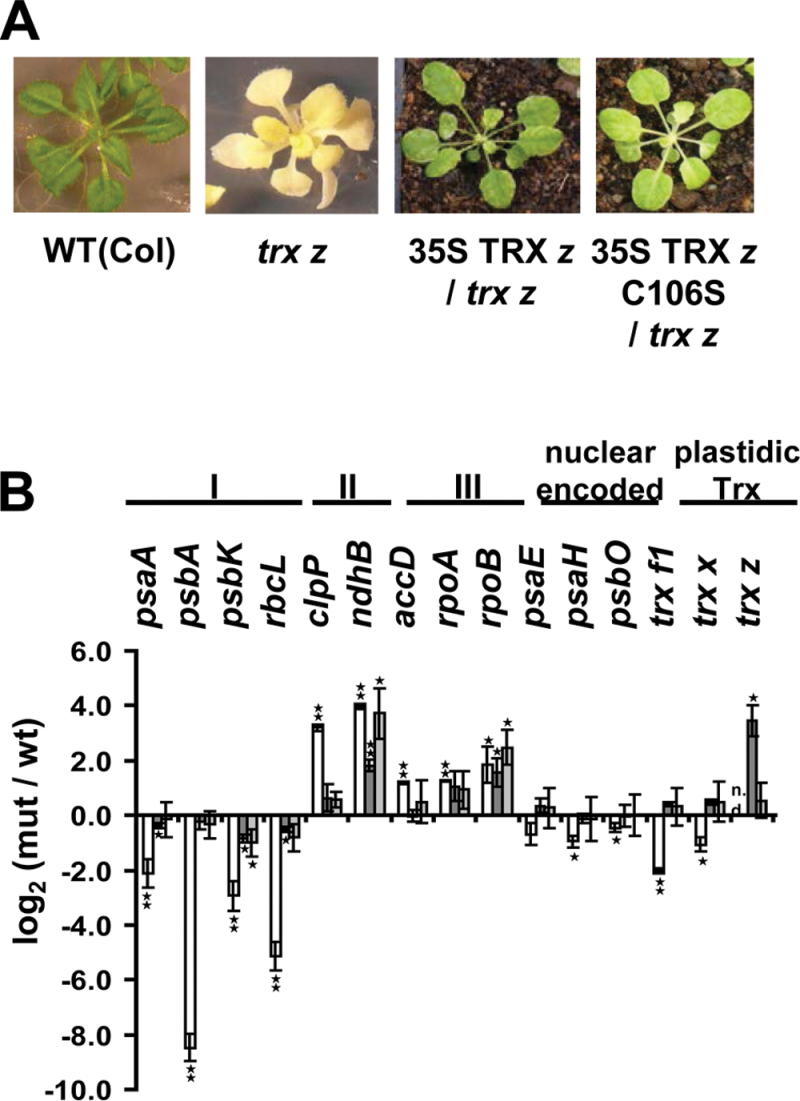
Phenotypic and transcriptional changes of *trx z* and complemented *trx z* plants compared to wild type (Col0). (A) Phenotype of *trx z* plants and complemented *trx z* plants. Plants were grown on MS medium (*trx z* plants) or soil (complemented *trx z* plants) for 3 weeks. (B) qRT-PCR analysis with gene-specific primers of plastid- and nuclear-encoded genes in *trx z* and complemented *trx z* plants. White, *trx z*; dark grey, 35S TRX *z*/*trx z*; light grey, 35S TRX *z* C106S/*trx z*. The log_2_ value (mutant/wild type) is given where 3.32 corresponds to a 10-fold upregulation and –3.32 to a 10-fold downregulation in the mutant or complemented plants, respectively, compared to the wild-type control. I, class I genes; II, class II genes; III, class III genes; data are mean and SD (*n*=3). 18S rRNA was used as a reference. Significant differences between *trx z*/complemented *trx z* plants and wild type (Col0) were calculated using Student’s t-test: *P<0.05, **P<0.01 (this figure is available in colour at *JXB* online).

Previous results indicate that the phenotype of *Arabidopsis trx z* knockout plants could be complemented by the introduction of the complete TRX *z* ORF under the control of the constitutive CaMV 35S promoter into the *trx z* background (Arsova *et al.*, 2010). In order to investigate whether the enzymic function of TRX *z* is essential for chloroplast function, *Arabidopsis trx z* plants were transformed to express the catalytically inactive TRX *z* C_106_S variant which is unable to form a mixed disulphide bridge with its target proteins and therefore has no redox-regulatory properties. As homozygous *trx z* plants are unable to grow autotrophically and do not develop any flowers, even not on sucrose-complemented agar, heterozygous plants were used for the transformation. Plants of the next generation were tested for homozygosity with respect to the insertion and successful expression of the complementation construct by PCR (Supplementary Fig. S1, available at *JXB* online). The phenotype of one representative homozygous BASTA-resistant T2-complemented line is shown in Fig. 2A. Complementation of the *Arabidopsis trx z* mutant line with a CaMV35S promoter-driven *TRX z* C_106_S construct rescued the *trx z* mutant phenotype and restored growth on soil. Therefore, it is concluded that TRX *z* redox activity is not essential for normal plant growth in *Arabidopsis*.

Some leaves of the complemented plants still displayed slight chlorosis at the leaf margins such as that previously observed for 35S:*TRX z*/*trx z* plants (Arsova *et al.*, 2010), suggesting that expression of the 35S promoter:*TRX z* cDNA fusion did not yield complete rescue of the *trx z* mutant phenotype in specific cells.

An expression analysis of selected plastome-encoded genes revealed that *Arabidopsis trx z* mutant lines exhibit transcriptional changes diagnostic for a perturbed PEP function (Arsova *et al.*, 2010). So-called class I genes, which are assumed to be exclusively transcribed by PEP, show drastically reduced transcript levels as compared to the wild-type control, while NEP-responsive class III genes are slightly upregulated. Class II genes, which can be transcribed by both polymerases, show a similar level of induction as class III genes (Arsova *et al.*, 2010). In order to determine whether the restoration of autotrophic growth in 35S:*TRX z* C_106_S-complemented *trx z* mutant plants would also result in a restoration of plastid gene expression to wild-type levels, qRT-PCR of the mRNA levels of selected class I, II, and III genes was performed. As shown in Fig. 2B, expression of the redox-inactive TRX *z* C_106_S variant was sufficient to re-establish expression of PEP-dependent class I genes. Class II and III genes still showed some upregulation in 35S TRX *z* C_106_S-complemented *trx z* plants as compared to the wild type. However, a similar level of expression of these genes was also observed in TRX *z*-complemented *trx z Arabidopsis* mutants. Thus, the effect on class II and III genes appears to be independent of the redox-activity of TRX *z*.

In essence, the redox activity of TRX *z* seems to be dispensable for both autotrophic growth of *Arabidopsis* and plastid gene expression under standard growth conditions.

### Photosynthetic parameters indicate restoration of PSII integrity in the 35S:*TRX z* C_106_S/*trx z* line

**Fig. 3.**
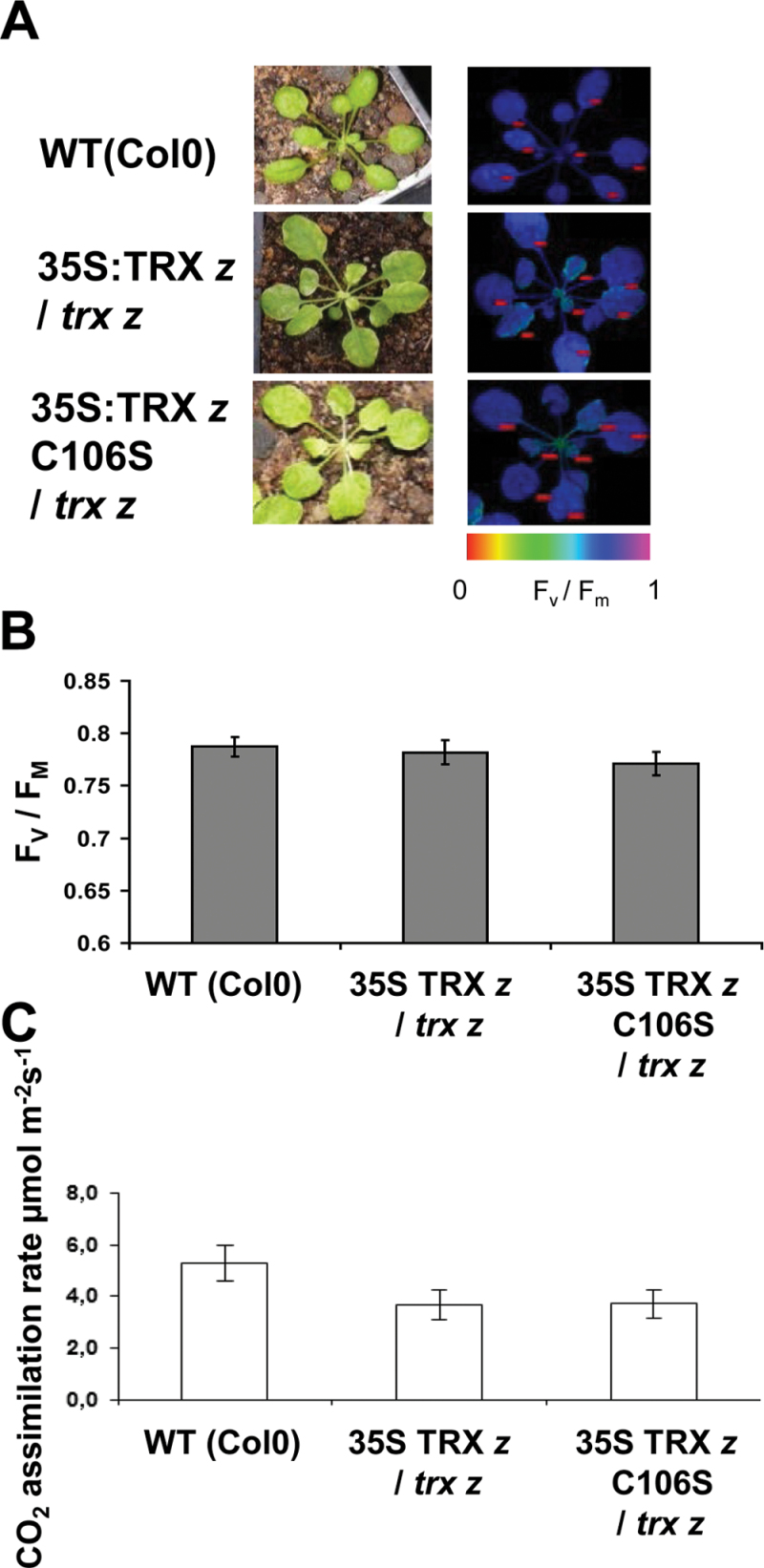
Measurement of photosynthetic parameters in complemented *trx z* plants. (A) Chlorophyll fluorescence was measured as an indicator of PSII integrity; representatives of complemented *trx z* plants are shown. (B) *F*_v_/*F*_m_ values of complemented *trx z* plants (five plants, 7–8 leaves each). (C) CO_2_ assimilation of complemented *trx z* plants (*n*=5).

Redox regulation of PEP plays a major role in the adjustment of plastid gene expression to fluctuations in light quantity and quality (Baginsky *et al.*, 1999; Steiner *et al.*, 2009). Although 35S:*TRX z* C_106_S-complemented plants were phenotypically indistinguishable from 35S:*TRX z* wild-type-complemented plants, a lack of TRX *z* redox activity could have subtle influence on plastid gene expression that could affect photosynthesis efficiency. Thus, to analyse the integrity of the two photosystems, this study determined the maximum PSII quantum yield by chlorophyll fluorescence measurement. The ratio of *F*_v_/*F*_m_ is an indicator for the efficiency of photosystem II photochemistry. As shown in Fig. 3A and B, no significant difference in the *F*_v_/*F*_m_ ratio could be observed between wild-type plants and the complemented lines, indicating that wild-type TRX *z* as well as the redox-inactive C_106_S variant are both sufficient to restore photosynthetic efficiency under standard growth conditions.

This was further corroborated by the fact that light-saturated photosynthesis at 400 μmol (mol CO_2_)^–1^ showed no difference between *trx z* plants complemented with either wild-type TRX *z* or the catalytically inactive TRX *z* C_106_S variant (Fig. 3C). The CO_2_ assimilation rate was slightly reduced in complemented plants as compared to the *Arabidopsis* wild type; however, this was independent of TRX *z* redox activity and thus likely to be an effect of the complementation *per se*.

### Redox regulation of FLN1 by TRX *z* is not essential for normal chloroplast development

**Fig. 4.**
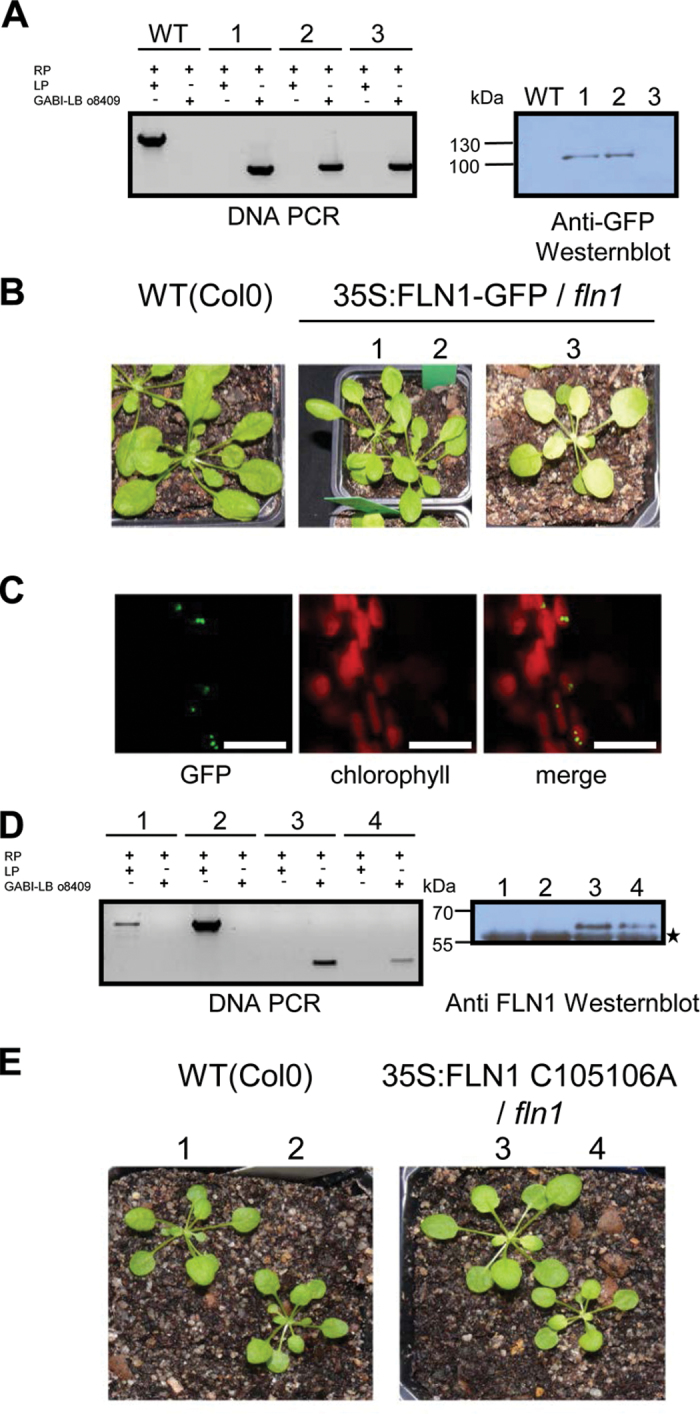
Molecular characterization of 35S:*FLN1*–GFP/*fln1* (A–C) and 35S:*FLN1*-C_105/106_S/*fln1* (D, E) plants. (A) Left panel: Analysis of genomic DNA by PCR shows a homozygous insertion in the *fln1* locus of complemented 35S:*FLN1*–GFP/*fln1* plants. Right panel: Western blot analysis of 35S:*FLN1*–GFP/*fln1* plants with a GFP-specific antibody. (B) Phenotype of complemented 35S:*FLN1*–GFP/*fln1* plants; plants were grown on soil for 3 weeks. (C) Subcellular localization of the *FLN1*–GFP protein in 10 35S:*FLN1*–GFP/*fln1* plants; green fluorescence of GFP (left) and chlorophyll auto fluorescence (centre) were detected separately by CLSM and both channels were merged (right); bar = 10 μm. (D) Left panel: Analysis of genomic DNA by PCR shows a homozygous insertion in the *fln1* locus of complemented 35S:*FLN1*-C_105/106_S/*fln1* plants. Right panel: Western blot analysis of 35S:*FLN1* C_105/106_S/*fln1* plants with an FLN1-specific antibody, asterisk indicates cross-reaction of the antibody with the large subunit of RubisCo. (E) Phenotypes of complemented 35S:*FLN1* C_105/106_S/*fln1* plants; plants were grown on soil for 3 weeks.

After having established that the redox activity of TRX *z* is dispensable for proper PEP function and autotrophic growth, this work turned its attention to FLN1, a target protein of TRX *z* that also constitutes an essential auxiliary factor of the PEP complex. Loss of *FLN1* by RNA interference or due to a T-DNA insertion in *Arabidopsis* leads to development of a typical PEP-deficient phenotype and the associated changes in plastid transcription (Arsova *et al.*, 2010; Steiner *et al.*, 2011; Gilkerson *et al.*, 2012). FLN1 contains two vicinal cysteine residues which mediate interaction with TRX *z* and are most likely responsible for redox modification *in vivo*. A mutation of a double-cysteine motif of the FLN1 protein to alanine (C_105/106_A) abolishes the interaction with TRX *z* (Arsova *et al.*, 2010). In order to investigate whether the redox-active C105/106 are required for FLN1 function *in vivo*, a previously identified *Arabidopsis* T-DNA insertion line (Steiner *et al.*, 2011; Gilkerson *et al.*, 2012) was transformed with the *FLN1* wild-type sequence and a *FLN1* C_105/106_A variant, respectively, and expressed under control of the constitutive CaMV 35S promoter. In order to analyse the subcellular localization of the FLN1 wild-type protein *in vivo*, a translational fusion to the N-terminus of green-fluorescent protein was generated. Due to the lack of autotrophic growth of the *fln1 Arabidopsis* mutant plants, the transformation was performed with *FLN1/fln1* heterozygous individuals. Plants of the next generation were tested for homozygosity with respect to insertion and expression of the transgene (Fig. 4A). As shown in Fig. 4B, the 35S promoter-driven *FLN1*–GFP construct was able to rescue the *fln1* mutant in a dose-dependent manner. Two lines with high *FLN1*–GFP expression level appeared as green as the wild type, while a line with barely detectable FLN1–GFP protein amount remained chlorotic when grown on soil. Confocal microscopy of leaves from *FLN1*–GFP-expressing plants revealed that the fusion protein is properly located to the chloroplast (Fig. 4C).

*Arabidopsis fln1* mutants complemented with a 35S:*FLN1* C_105/106_S construct were also able to grow autotrophically on soil (Fig. 4D and E), indicating that a redox modification of FLN1 by TRX *z* is not essential for chloroplast development and thus for autotrophic growth.

**Fig. 5.**
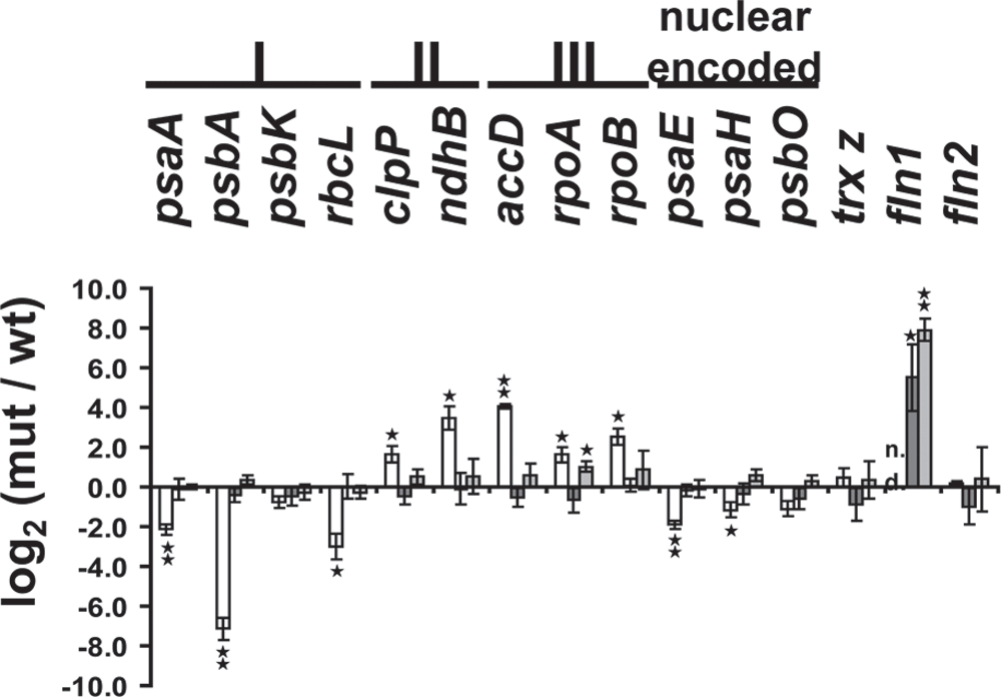
Changes in transcript abundance of plastid-encoded and nuclear-encoded genes in *fln1* and complemented *fln1* plants. Quantitative RT-PCR analysis with gene-specific primers of plastid-encoded and nuclear-encoded genes in *fln1* and complemented *fln1* plants. White, *fln1*; dark grey, 35S:*FLN1*–GFP/*fln1*; light grey, 35S:*FLN1* C_105/106_S/*fln1*. The log_2_ value (mutant/wildtype) is given where 3.32 corresponds to a 10-fold upregulation and –3.32 to a 10-fold downregulation in the mutant or complemented plants compared to the wild-type control. I, class I genes; II, class II genes; III, class III genes; data are mean and SD (*n*=3). 18S rRNA was used as a reference. Significant differences between *fln1*/complemented *fln1* plants and wild type (Col-0) were calculated using Student’s t-test: *P<0.05 and **P<0.01.

Next, this study analysed the gene expression pattern of chloroplasts in complemented *fln1* plants by qRT-PCR. As previously described (Gilkerson *et al.*, 2012), *fln1 Arabidopsis* plants show specific alterations in the plastidic transcription pattern such as those observed in *trx z* or other *ptac* mutants. It could be shown that complementation with FLN1–GFP as well as with FLN1 C_105/106_S both restored PEP-dependent transcription in plastids (Fig. 5). This led to the conclusion that redox regulation of FLN1 is not essential for normal function of PEP during chloroplast development.

### *TRX z* and *FLN1* are predominantly expressed in young developing leaves during vegetative growth

To characterize the *in planta* expression pattern of *TRX z* and *FLN1* at high spatial resolution, wild-type *Arabidopsis* plants were transformed with constructs containing the β-glucoronidase (GUS) reporter gene transcriptionally fused to the respective promoter region. A sequence of approximately 1.5kb upstream of the start codon of each gene was amplified by PCR to cover the complete regulatory elements of the promoter region. The resulting transgenic plants were stained for GUS activity in different tissues and at various developmental stages. The following observations have been made on multiple lines of the relevant transformants.

**Fig. 6.**
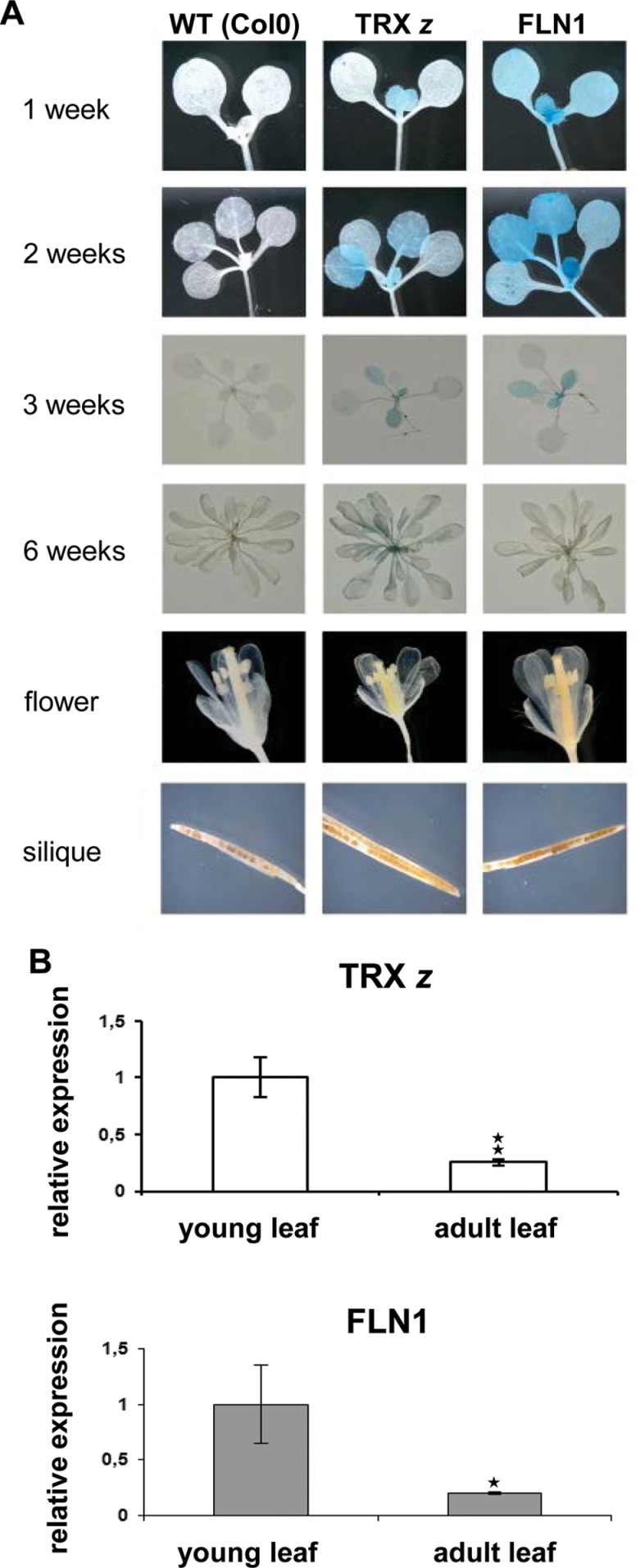
Expression analyses of *TRX z*, *FLN1*, and *FLN2* in *Arabidopsis*. (A) Analysis of gene-specific promoter activity by using promoter–GUS fusion constructs. *Arabidopsis* plants were transformed with promoter-GUS constructs by floral dipping. Positive transformants were grown on selective medium and subsequently transferred to soil. GUS expression at different growth stages and in different tissues was detected by GUS activity staining. (B) qRT-PCR analysis with gene-specific primers of tissue-specific expression of TRX *z*, *FLN1*, and *FLN2* in young and mature *Arabidopsis* leaves. Data are mean and SD (*n*=3). 18S rRNA was used as a reference. Significant differences between young and adult leaves were calculated using Student’s t-test: *P<0.05 and **P<0.01.

In 1-week-old seedlings, GUS staining was observed in the first true leaves in both reporter lines (Fig. 6A). The cotelydons remained largely unstained or were only faintly stained as in case of pFLN1:GUS plants. The second true leaves in 2-week-old plants also showed GUS expression; however, at 3 weeks, GUS staining in the oldest leaves appeared to be diminished and it was absent in leaves of 6-week-old plants (Fig. 6A). Taken together, the GUS staining indicates that expression of all genes occurs predominantly in early developmental stages of the leaf and mature leaves seem to show no promoter activity. No GUS staining was observed in flowers or siliques (Fig. 6A).

To further corroborate these findings, a qRT-PCR analysis of *TRX z* and *FLN1* expression was conducted on RNA extracted from young and mature leaves, respectively. The results confirmed a sharp decline in expression of both genes during leaf development (Fig. 6B).

## Discussion

During the onset of photomorphogenesis, the *E. coli*-like PEP is reorganized into a much more complex eukaryote-like PEP enzyme complex. This involves the association of a range of additional nuclear-encoded subunits which are believed to impart novel regulatory properties to the PEP complex in order to accommodate the physiological conditions and requirements of the mature chloroplast (Pfannschmidt *et al.*, 2000). A recent proteomics study identified a total number of 10 auxiliary proteins that appear to be tightly associated with the PEP complex in chloroplasts (PEP-associated proteins, PAPs) and based on their predicted biochemical function, these were divided into two groups (Steiner *et al.*, 2011). The first group comprised members whose function is likely related to gene expression or transcription while the second group consists of proteins involved in redox-dependent processes or regulation. Two members of this second group, TRX *z* and FLN1, were previously shown to interact with each other in a thiol-dependent manner (Arsova *et al.*, 2010). The substitution of C_106_ to S within the TRX *z*-active site not only abrogates its redox activity but also almost completely abolishes binding to FLN1 in yeast, which is consistent with the proposed TRX reaction mechanism. In turn, a C_105/106_A substitution within the FLN1 polypeptide abrogates binding to TRX *z* (Arsova *et al.*, 2010). This indicates that TRX *z* redox-modulates FLN1 function *in vivo*, making the TRX *z*/FLN1 redox couple a likely candidate for translating redox signals into changes in PEP activity.

A knockout in either *TRX z* or *FLN1* leads to the inability to grow autotrophically. Mutant plants develop pale-white leaves and only survive on sucrose-complemented medium (Arsova *et al.*, 2010; Schröter *et al.*, 2010; Steiner *et al.*, 2011; Gilkerson *et al.*, 2012). This indicates that both proteins play an essential role for PEP function. The plastidial TRX system is generally assumed to be genetically redundant as the deficiency of single isoforms only leads to mild phenotypic changes in a range of physiological processes (Benitez-Alfonso *et al.*, 2009; Courteille *et al.*, 2013; Laugier *et al.*, 2013; Thormahlen *et al.*, 2013). Recently, the simultaneous inactivation of three *m*-type TRXs in *Arabidopsis* was shown to result in pale-green leaves as a consequence of a disturbed photosystem II assembly in these plants (Wang *et al.*, 2013). Thus, lack of the redox-regulatory function of particular TRXs can have a strong impact on plant phenotype. In case of *TRX z* knockout plants, it is not clear whether the strong phenotype arises from a loss of redox regulation of PEP or of another function of this particular TRX *z* isoform. Given the fact that the *E. coli*-like PEP in etioplasts does not require TRX *z* for activity, it appears possible that redox activity of TRX *z* is not essential for PEP function in chloroplasts. Complementation of *trx z* mutant plants with a redox-inactive TRX *z* C_106_S variant fully restores plastid gene expression, photosynthetic activity, and growth on soil, strongly suggesting that loss of redox regulation of PEP is not the cause for the drastic phenotype of the *trx z* plants. In fact, none of the PAPs seems to possess a function that is truly essential for transcription but they all share the same strong mutant phenotype when the corresponding gene is inactivated (Steiner *et al.*, 2011). This has been explained by a disturbance of correct RNA polymerase complex formation during an early phase of plastid development in PAP mutant plants. Accordingly, if one of the PAPs is lacking, either the generation of the chloroplast PEP is interrupted or the intermediate complex is unstable, resulting in a complete block of PEP activity (Pfalz and Pfannschmidt, 2013). This is further corroborated by the complementation of the *fln1* knockout with a FLN1 C_105/106_A double-mutant protein, which fully restores plastid transcription and autotrophic growth. Although the biological activity of FLN1 is currently unknown its redox regulation by TRX *z* appears to be dispensable for PEP function under standard growth conditions. The C_105/106_A double mutation completely abolishes the ability of FLN1 to interact with TRX *z* in yeast (Arsova *et al.*, 2010); however, *in planta* this mutation does not seem to prevent TRX *z* from being properly assembled into the PEP complex, otherwise the phenotype could not be restored. This suggests that FLN1 might interact with PAPs other than TRX *z* or perhaps with nucleic acids. Recently, it was shown that FLN1 interacts with pTAC7/PAP12 in yeast (Yu *et al.*, 2013); however, this interaction awaits confirmation *in vivo*.

The same is true for TRX *z*, which needs to find other interaction partners in *FLN1* C_105/106_A-complemented plants in order to be assembled into the PEP complex. One likely candidate would be FLN2 which is closely related to FLN1 and also a TRX *z* target protein (Arsova *et al.*, 2010). Although FLN2 has been found to be a component of PEP in some experiments (Steiner *et al.*, 2011), it appears not to be a core PAP (Steiner *et al.*, 2011). Inactivation of FLN2 does not have drastic phenotypic effects (Gilkerson *et al.*, 2012), indicating that FLN1 and FLN2 are not functionally equivalent. Furthermore, attempts to complement *fln1* mutant plants with a 35S:*FLN2* construct have so far failed (data not shown), also suggesting different functions of the two proteins. TRX *z* has recently been reported to interact with AtECB1/MRL7, a protein containing a thioredoxin-like fold that is also required for PEP function although it does not appear to be a core component of the PEP complex (Qiao *et al.*, 2011; Yua *et al.*, 2013). Thus, even if TRX *z* is unable to interact with FLN1 C_105/106_S it could still retain its structural function for the PEP complex through the interaction with AtECB1/MRL7.

It has previously been proposed that assembly of the eukaryote-like PEP complex represents a developmental bottleneck during early chloroplast development that leads to abortion of proper chloroplast biogenesis if disturbed (Pfalz and Pfannschmidt, 2013). This would require a concerted expression of PAP encoding genes during the transition from etioplasts to chloroplasts prior to the establishment of the photosynthetic apparatus. Accordingly, it has been shown that the *Arabidopsis fln1* mutant can be rescued by a dexamethasone-inducible wild-type gene copy only when the expression of the construct is induced within the first 24h after germination (Gilkerson *et al.*, 2012). That said, promoter–GUS analyses indicated that *TRX z* and *FLN1* are expressed in young tissues with decreasing expression during leaf maturation. Notably, 35S:*FLN1*-complemented *fln1* plants developed chlorotic leaves at maturation, suggesting a detrimental effect of elevated FLN1 levels at later stages of development (data not shown).

In conclusion, complementation of *trx z* and *fln1* knockout plants by the respective redox-inactive protein variant demonstrates that a lack of redox regulation of PEP does not lead to phenotypic changes under normal growth conditions. These data support a model in which proper assembly of the PEP complex during plastid development is an essential early step during chloroplast biogenesis and its disturbance cannot be reversed if a certain time point in the programme has been passed (Pfalz and Pfannschmidt, 2013). Given the high degree of conservation of the TRX *z*/FLN1 redox couple within the plant kingdom, it seems likely that redox regulation of PEP function through TRX *z* plays a role during adaptation to changes in light quality or quantity. The transgenic *Arabidopsis* lines complemented with redox-inactive variants of TRX *z* and FLN1, respectively, provide a useful tool to study adaptation responses of chloroplast transcription under fluctuating light conditions.

## Supplementary material

Supplementary data are available at *JXB* online.

Supplementary Fig. S1. Molecular analysis of the *trx z Arabidopsis* T-DNA insertion mutant complemented with CaMV 35S:*TRX z* C_106_S expression construct.

Supplementary Table S1. Primers used in this study.

Supplementary Data
