# Supplementary material for: Redox activity of thioredoxin z and fructokinase-like protein 1 is dispensable for autotrophic growth of Arabidopsis thaliana
Source: J Exp Bot. 2014 Mar 22;65(9):2405–13. doi: 10.1093/jxb/eru122 (PMC4036507; doi:10.1093/jxb/eru122)
Supplement: Supplementary Data [file supp_65_9_2405__index.html]

Redox activity of thioredoxin z and fructokinase-like protein 1 is dispensable for autotrophic growth of Arabidopsis thaliana — Redox activity of thioredoxin z and fructokinase-like protein 1 is dispensable for autotrophic growth of Arabidopsis thaliana — Supplementary Data 

# Redox activity of thioredoxin *z* and fructokinase-like protein 1 is dispensable for autotrophic growth of *Arabidopsis thaliana*

## Supplementary Data

Data files

**Files in this Data Supplement:**

- Supplementary Data - Supplementary Data
