# Supplementary material for: Redox activity of thioredoxin z and fructokinase-like protein 1 is dispensable for autotrophic growth of Arabidopsis thaliana
Source: J Exp Bot. 2014 Mar 22;65(9):2405–13. doi: 10.1093/jxb/eru122 (PMC4036507; doi:10.1093/jxb/eru122)
Supplement: Supplementary Data [file supp_eru122_jexbot120238_file001.pdf]

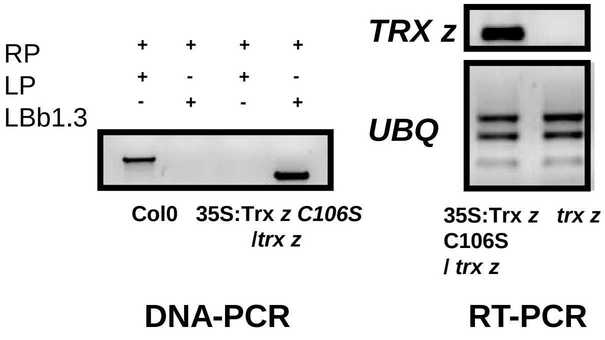

Figure S1: Molecular analysis of the *trx z Arabidopsis* T-DNA insertion mutant complemented with a CaMV 35S: Trx *z* C106S expression construct (B).

Left: PCR on genomic DNA. RP and LP result in amplification of the wild type allele in Col0 control plants while RP and the T-DNA specific primer LBb1.3 give no signal. In 35S:Trx *z* C106S/ *trx z* plants no wild type allel can be amplified while the T-DNA can be detected, indicating a homozygous *trx z* background. Right: RT-PCR proves expression of the 35S:Trx *z* C106S transgene in the *trx z* background.

**Supplemental Table 1: Nucleotide sequences of gene-specific primers.**		
Nucleotide sequence of gene-specific primers for construction of the TRX *z E. coli* expression construct		
Trx z His_6	Fwd	5’- GGATCCCATCATCATCATCATCATGGCAAGTTTGTC AGAGAA - 3’
	Rev	5’- GTCGACTCACATCTCGTTGTCAATGATATC- 3’
Nucleotide sequence of gene-specific primers used to confirm T-DNA insertion **(**Salk 028162)		
028162_LP		5’-CCAACTACGCGACAAGGTATC-3’
028162_RP		5’-TTTTCCACACCTCAACACTCC-3’
LBb1.3		5'-ATTTTGCCGATTTCGGAAC-3'
trx z	Fwd	5’-GCAGTAGAGTATGAGAGCAATGCA-3’
	Rev	5’-CATCTCGTTGTCAATGATATCGTG-3’
ubq	Fwd	5'-ATGCAGATYTTTGTGAAGAC-3'
	Rev	5'-ACCACCACGRAGACGGAG-3'
Nucleotide sequence of gene-specific primers for used to confirm T-DNA insertion (GABI-Kat 443A08)		
443A08_LP		5’-CAAATAGCGAGTCCTCAGGTG-3’
443A08_RP		5’-GATCAATTCCCACAAGGAAGC-3’
o8474		5’-ATAATAACGCTGCGGACATCTACATTTT-3’
fln1	Fwd	5’-CAAGATCAAGTTTAAGGACGG-3’
	Rev	5’-GTTCCATCAGTCACAACCAG-3’
Nucleotide sequence of gene-specific primers for construction of the 35S:TRX z vector		
TRX z cDNA	Fwd	5’-GGATCCGGCAAGTTTGTCAGAGAAG-3’
	Rev	5’-TCACATCTCGTTGTCAATGATATC-3’
Nucleotide sequence of gene-specific primers for construction of the 35S:FLN1 vector		
FLN1 cDNA	Fwd	5’-CACCATGGCTTCACTTCTTATTTTCC-3’
	Rev	5’-TGCCCACATTGATGGAACATAAACTTG-3’
Nucleotide sequence of gene-specific primers used for site-directed mutagenesis		
FLN1 C105A/C106A	Fwd	5’-ACGACGATCCGCCTCTTGTGGCTGCCTTCGGAGCTGTACAGAAGGA-3’
	Rev	5’-TCCTTCTGTACAGCTCCGAAGGCAGCCACAAGAGGCGGATCGTCGT-3’
TRX z C106S	Fwd	5’-GATTTTTATGCGACATGGAGTGGACCTTGTATCTTG-3’
	Rev	5’-CAAGATACAAGGTCCACTCCATGTCGCATAAAAATC-3’
TRX z C109S	Fwd	5’-CATGGTGTGGACCTAGTATCTTGATGGCCCAG-3’
	Rev	5’-CTGGGCCATCAAGATACTAGGTCCACACCATG-3’
Nucleotide sequence of gene-specific primers used for qPCR and RT-PCR		
psaA At	Fwd	5'-CTACTTTGCCACCCACTGC-3'
	Rev	5'-TGAGTGCTTTAGGGCGTCC-3'
psbA At	Fwd	5'-GCATAGCACTGAATAGGGAGCCG-3'

	Rev	5'-GCGACCTTGGATTGCTGTTGC-3'
psbK At	Fwd	5'-AGTCGCCAAATTGCCAGAGG-3'
	Rev	5'-AGCTTGCCAAACAAAGGCTAA-3'
rbcL At	Fwd	5'-TTGGCAGCATTCCGAGTAACTCCT-3'
	Rev	5'-CTGGTAAGTCCATCGGTCC-3'
clpP At	Fwd	5'-GGAGGAGCAATTACCAAACG-3'
	Rev	5'-GCTTGGGCTTCTGTTGCTGAC-3'
ndhB At	Fwd	5'-GGTCTAATGAGGCTACTATG-3'
	Rev	5'-CAAGAGAAACCATGAACCAGA-3'
accD At	Fwd	5'-GAAGGTTCACAAGCGGCTG-3'
	Rev	5'-GAAATAACTCGCTCAGAACAC-3'
rpoA At	Fwd	5'-TGCGATGCGAAGAGCTTTAC-3'
	Rev	5'-CAATGATTTCCACAGCGGG-3'
rpoB At	Fwd	5'-GCGAAAGAATCCTCCTATGC-3'
	Rev	5'-CCACCTCACATCAATAACTC-3'
psaE At	Fwd	5'-CGTGTCTTTCTTGCCGATGA-3'
	Rev	5'-TGGGTTTGGTGGCAGTAGC-3'
psaH At	Fwd	5'-GCTAATGGTGTGGTGGCTAA-3'
	Rev	5'-GGATTGTAAGGAGAAGGAGC-3'
psbO At	Fwd	5'-CAGCCTCTCTCCAATCCAC-3'
	Rev	5'-GAGGTGGCAAGAGCGAATC-3'
trxf1 At	Fwd	5'-GTGACGGAGGTCGATAAGGA-3'
	Rev	5'-AATGGCCGGTTATCTGGATT-3'
trxx At	Fwd	5'-CTAACCGCCACCTGCTTTC-3'
	Rev	5'-GCACCAAGTAGCGACGAAT-3'
trxz At	Fwd	5'-TTATGCGACATGGTGTGGAC-3'
	Rev	5'-CCCTTCTGTCCTGATTGCAT-3'
fln1 At	Fwd	5'-CGCCATTCACAAGCATCAAGG-3'
	Rev	5'-CTTCTTCTTCCTCCCTTTGC-3'
fln2 At	Fwd	5'-ACCGAGAAGAAAGTGAGACG-3'
	Rev	5'-CATCACTAACCTCAGCATCC-3'
18S rRNA At	Fwd	5'-AAACCCCGACTTATGGAAGG-3'
	Rev	5'-CGAACCCTAATTCTCCGTCA-3'
Nucleotide sequence of gene-specific primers used for generation of GUS-promoter constructs		
Trx z promoter	Fwd	5’-CACCCAGTTTGGAGGGAATCAAGCTCC-3’
	Rev	5’-GGATCCTTTGGAAATCGATTTCTGCTTCAAATG-3’
FLN1 promoter	Fwd	5’-CACCATGTCCAGAAGCCCATTCTTTTCTG-3’
	Rev	5’-TCCTGATGGGTTTTTGACACCACTGTG-3’

FLN2 promoter	Fwd	5’-CACCGAACTTGTTGTGTTATCCAAGTACC-3’
	Rev	5’-GGATCCCAAATAAGAGCTCTTTTCCAAACG-3’
